# The Genes of Antimicrobial Peptides for the Therapy of Intracellular Infections

**Published:** 2009-04

**Authors:** V. N. V. N. Lazarev

**Affiliations:** 1 Scientific Research Institute of Physical-Chemical Medicine, ul. Malaya Pirogovskaya 1a, Moscow, 119992, Russia

## 

Resistance to antibiotics is of great social and economic importance and is regarded as a threat to the national security of any country and the global community as a whole. Among the bacterial agents of different infections, resistance to some antibiotics can reach 98%. Infections caused by antibiotic-resistant strains are distinguished by their significant duration, they often require hospitalization, they increase the length of hospital stay, and they often worsen the prognosis for a disease [1]. If the chosen medicines turn out to be ineffective, the doctors have to use second- or third-order medicines, which are often rather expensive, less safe, and not always available. All these facts increase direct and indirect economic expenditures, as well as cause a risk of antibiotic-resistant strain propagation. Causative agents of intracellular infections such as mycoplasmas and chlamydiae are characterized by high antibiotic resistance. Treating mycoplasmosis and clamidiosis with a wide range of antibiotics is almost ineffective due to the quick formation of resistance to these medicines, and, as a result, the development of virus persistence in the organism. 

In connection with this, it is essential to create alternative therapeutic agents which will not cause or limit antibiotic resistance. Antimicrobial peptides (AMPs) may be such therapeutic agents. They represent a unique and quite diverse group of compounds which make up a major component of the natural immunity of all organisms [2]. Compared to antibiotics, antibacterial peptides have the following advantages: a wider range of antibacterial action, functional activity at micromolar concentrations, the absence of virus resistance to antimicrobial peptides, and the synthesis capability of natural peptide analogues with altered biological properties. The causative agent cannot become resistant to AMPs because of the unique mechanism of their action, which consists in the formation of channels and the following fragmentation of the bacterial cell membrane. However, to date, all investigations devoted to the study of AMPs have focused on exogenic (synthesized) peptides, while the mechanism of AMPs synthesized directly in the infected cell is still unclear. We chose melittine as a model peptide, which is an amphipathic α-helical peptide (a major component in bee poison) [3]. 

In that review, we were the first to show the inhibition of such experimental infections as mouse Mуcoplasma hominis and Chlamydia trachomatis and broiler chicken Mycoplasma gallisepticum.

In this review, we used a pBI/mel2/rtTA plasmid vector containing the melittine gene under the control of the tetracycline-dependent CMV promoter and the transacting rtTA protein gene controlled by the early constitutive CMV promoter [4].

Using this plasmid construction allows the expression level of the antimicrobial peptide genes in the organism to be accurately regulated with the help of different inducer doses, which is of great importance when the products of the expressed genes are toxic. 

We used female mice of the BALB/c line (6-8 weeks old and weighing 18-22 g). 

Before contamination with M.hominis, the mice were injected subcutaneously with estradiol (Intervet UK, Great Britain) in doses of 0.5 mg per mouse (0.1 ml four times, with a week interval). Progesterone (Depo-Provera, Great Britain) was injected subcutaneously in a dose of 2.5 mg per mouse (0.1 ml, four days before contamination with C. trachomatis).

M. hominis suspension (109 cell/ml titer) was injected (50 mcl) into the mice intravaginally after the second estradiol injection. A fraction of C. trachomatis elementary bodies (106 IFU/ml titer; IFU, inclusion-forming unit) was injected into the mice intravaginally (50 mcl) after progesterone injection. Recombinant pBI/mel2/rtTA plasmid vector was injected intravaginally using the Effectene Transfection Reagent (Qiagen GmbH, Germany). The recombinant vector was injected twice: 24 h before infection with M. hominis or C. trachomatis and 14 days after infection in doses of 2 μg per DNA/mouse (25 mcl) with addition of 25 mcl of cacao oil to increase the suspension viscosity. Doxycycline hydrochloride (ICN Pharmaceuticals, Moscow, Russia) was used as inducer of melittine gene transcription. The medicine was injected intramuscularly into the mice infected with M. hominis and C. trachomatis in doses of 2 μg per mouse and 1 μg per mouse, respectively, (50 mcl) at the moment of vector injection. 

The animals were subdivided into three groups (six mice in each group, two independent experiments). Group 1 was infected with M. hominis or C. trachomatis without pBI/mel2/rtTA plasmid vector or doxycycline. Group 2 was injected with doxycycline in the corresponding dose with the following infection of M. hominis or C. trachomatis. Group 3 was injected with pBI/mel2/rtTA plasmid vector and doxycycline followed by M. hominis or C. trachomatis.

To determine the M. Hominis titer after the pBI/mel2/rtTA plasmid vector injection, we prepared ten-fold diluted lavages from the upper urogenital tracts of the mice. To determine the C. trachomatis titer, we used the direct fluorescence reaction and infected the McCoy cell line with the vaginal lavages. 

**Fig. 1.**
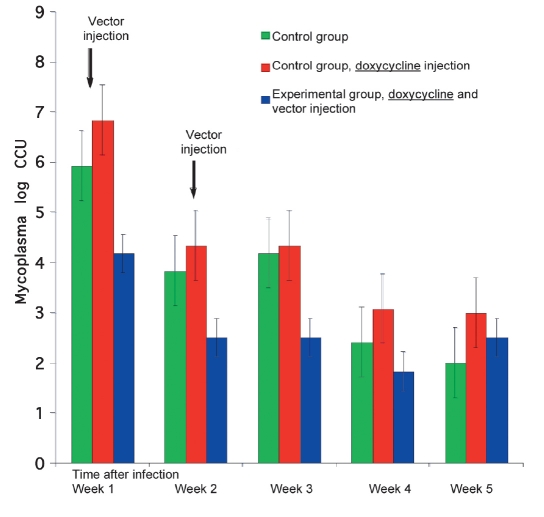
Influence of the recombinant pBI/mel2/rtTA plasmid vector injection on the M. hominis content in the vaginas of mice infected.

The injection of the recombinant pBI/mel2/rtTA vector and the following contamination of mice were finished by the M.hominis infection inhibition. The results may be seen in Fig. 1. The M. hominis titer in the vaginal lavages of Group 1 mice varied, decreasing from 5.9 to 2.4 log10 ccu/ml (сcu, color change unit) in four weeks. In the Group 3 mice, which were injected with the recombinant pBI/mel2/rtTA vector and doxycycline before infection, the M. hominis titer was within 4.1-1.8 log_10_ ccu/ml. 

In the case of the pBI/mel2/rtTA plasmid vector injection and the following infection of mice with С. trachomatis, the infection inhibition level was 45-80% (Table 1). 

**Table 1:** Influence of the recombinant pBI/mel2/rtTA plasmid vector injection on the C. *trachomatis* content in the vaginas of mice infected.

Observation pe-riod	C. trachomatis titer in vaginal lavages of mice infected (number of C. trachomatis inclusions/ml)
	2 days	6 days	9 days	13 days	16 days	20 days	27 days
Group 1 12950	8490	4250	5220	2510	1070	1570
Group 2	12600	9750	3930	4850	2140	980	1470
Group 3	6850	2710	1920	2090	1080	370	350

Note: Differences between Group 3 and Groups 1 and 2 are reliable (P < 0.05).

In spite of the fact that we did not achieve complete recovery of the mice from mycoplasmas and chlamydiae in the observation period, the rate of causative agent elimination was higher in Group 3 than in the control groups. Three mice of Group 3 infected with M. hominis recovered from the virus on the 21st day after infection; in the control groups 1 and 2, all mice had M. hominis. As for the mice infected with С. trachomatis, four mice from Group 3 were free from the virus on the 27th day after infection.

It should be noted that we did not obtain reliable statistical differences in the titers of mice from Groups 1 and 2 infected with M. hominis or С. Trachomatis, which, firstly, testifies to the absence of an uncontrolled expression of the melittine gene, and, secondly, to the fact that the chosen inducer (doxycycline) concentration does not influence the infection process development. 

To investigate the influence of recombinant vector injection on the development of the Mycoplasma gallisepticum infection, 60 21-day-old Ross broiler chickens were marked and subdivided into four groups consisting of 15 chickens in such a way that the chickens’ average weight was analogous in each group on the basis of the Student t-test.

Group 1 was not infected with M. gallisepticum or injected with the recombinant pBI/mel2/rtTA plasmid vector. Group 2 was infected with M. gallisepticum, but the recombinant pBI/mel2/rtTA plasmid vector was not injected. Group 3 was injected with the plasmid vector 5 h before infection with M. gallisepticum. Moreover, the mentioned chickens were injected intramuscularly with doxycycline (ICN Pharmaceuticals, Moscow, Russia)-which acted as an inducer of melittine gene transcription-24 and 5 h before the infection in doses of 0.1 per chicken (in the volume of 100 mcl). Group 4 was injected with doxycycline (in the same dose and with the same intervals), followed by infection with M. gallisepticum. Chickens of that group were not injected with the pBI/mel2/rtTA plasmid vector. 

All chickens were subject to clinical, postmortem, immunologic, and biological examinations. 

Nine days after infection, the Groups 1 and 3 did not have any respiratory symptoms. At the same time, Groups 2, 4, and 5 were revealed to have respiratory rale. The second group was characterized by a reliable statistical decrease in average weight. M. gallisepticum extraction from the chickens’ internal organs is of special interest (Table 2). 

**Table 2:** M. gallisepticum extraction from different parts of respiratory tract and internal organs.

Organ	Group 1	Group 2	Group 3	Group 4
Respiratory tract	Windpipe	0/14 ^a^	14/14	14/14	14/14
	Air pockets	0/14	12/14	10/14	14/14
	Lungs	0/14	10/14	6/14	11/14
	Total quantity of reisolations	0	36 ^b^	30	39
Internal organs	Liver	0/14	4/14	0/14	3/14
	Milt	0/14	3/14	0/14	4/14
	Kidneys	0/14	8/14	6/14	7/14
	Heart	0/14	3/14	0/14	3/14
	Total quantity of reisolations	0	18	6 ^c^	17

^a^ Quantity of mycoplasma reisolations/total number of chickens ^b^ Differences between Group 2 and Groups 3 and 4 are not statistically reliable. ^c^ Differences between Group 3 and Groups 2 and 4 are statistically reliable, P ≤ 0.01.

In spite of the fact that we did not obtain reliable differences in the frequency of M. gallisepticum reisolation from the chickens’ respiratory tracts in Groups 2 and 3 (Table 2), M. gallisepticum was detected only in 6 out of 56 internal samples. The livers, spleens, and hearts of that group of chickens did not contain M. gallisepticum.

Undoubtedly, the most important mechanism of membrane-active antimicrobial peptides, which leads to the inhibition of mycoplasmosis and clamidiosis infections in the cell culture and in vivo, is their direct cytotoxic action on these bacteria [5]. 

Moreover, the in vitro processing of mycoplasmas with amphipathic peptides such as cecropin A, melittine, and magainin 2 depolarizes their plasmamembranes, alters their morphology, and decreases their mobility [6]. As was shown previously, the melittine gene expression in the HeLa cell culture results in a reduction of the transmembrane potential of the transfected cell [7], which is followed by a breakdown in the process of mycoplasma and chlamydia adhesion in the cell and, as a consequence, an interruption of the normal cycle of their development [8]. Moreover, it is quite possible that melittine expression alters the cell's cytoskeleton, and, as a consequence, breaks down the traffic of chlamydia inclusions. 

In spite of the fact that we did not manage to completely eliminate the virus from the urinogenital and respiratory tracts in our experiments, these data allow us to suggest that the recombinant plasmid vectors expressing the antimicrobial peptide genes may be considered as potential agents for preventing and treating micoplasmosis and clamidiosis.
